# Overview of biophysical approaches to study osteogenic proteins in lipidic interfaces

**DOI:** 10.1007/s12551-026-01455-6

**Published:** 2026-09-08

**Authors:** Larwsk Hayann, Maryanne T. Mello, Juçara G. Cominal, Iara S. Lima, Luiz H. S. Andrilli, João Paulo M. Machado, Juliana R. Silva de Sousa, Pedro Alexandre B. S. Azevedo, Mariana F. de Oliveira, Ana Paula Ramos, Pietro Ciancaglini

**Affiliations:** Department of Chemistry, Ribeirão Preto Faculty of Philosophy, Sciences, and Letters at Ribeirão Preto, University of São Paulo, Ribeirão Preto, São Paulo Brazil

**Keywords:** Lipid–protein interactions, Biomimetic membranes, Extracellular vesicles, Biophysical techniques

## Abstract

Bone biomineralization is a tightly regulated process mediated by osteogenic proteins associated with extracellular vesicles known as matrix vesicles (MVs), which function as specialized nanoreactors for the nucleation and propagation of biological apatite. The activity of MV-associated proteins, including tissue-nonspecific alkaline phosphatase (TNAP), nucleotide pyrophosphatase/phosphodiesterase 1 (NPP1), PHOSPHO1, and annexins, is strongly influenced by the physicochemical properties of the vesicular membrane. Membrane composition, lipid packing, phase behavior, surface charge, and lipid microdomains regulate protein localization, conformation, catalytic activity, and intermolecular interactions, thereby controlling mineral formation. However, the structural complexity and heterogeneity of native MVs have hindered a detailed mechanistic understanding of protein–lipid interactions during biomineralization. This review examines the biophysical principles governing lipid membrane organization and their influence on the function of membrane-associated osteogenic proteins. Emphasis is placed on biomimetic membrane systems, including liposomes, proteoliposomes, giant unilamellar vesicles, Langmuir monolayers, and Langmuir–Blodgett films, which provide simplified and controllable platforms for investigating protein–lipid interactions and membrane-mediated mineralization. We also summarize key techniques for characterizing native vesicles and biomimetic membranes, including atomic force microscopy, confocal and electron microscopy, dynamic light scattering, nanoparticle tracking analysis, flow cytometry, Fourier-transform infrared and Raman spectroscopy, and boron-doped diamond microelectrodes. Finally, advanced and complementary approaches such as surface plasmon resonance, small-angle X-ray scattering, and microfluidic platforms are highlighted for their potential to advance the molecular understanding of membrane-associated biomineralization. Collectively, these complementary models and analytical methods provide an integrated framework for elucidating matrix vesicle function and guiding the rational design of biomaterials and vesicle-inspired therapeutic strategies for bone regeneration.

## Cellular and molecular basis of bone formation

Bone is a specialized connective tissue composed of a mineralized matrix that provides mechanical strength and protection to the body. Its matrix contains biological apatite crystals [Ca₁₀(PO₄)₆(OH)₂], which are responsible for bone rigidity and serve as a reservoir for calcium and phosphate (Grabowski 2015). In addition to its mineral phase, bone possesses an organic matrix mainly composed of type I collagen, along with smaller amounts of other collagen types such as III, V, XI, and XIII. Together, collagens represent approximately 90% of the organic matrix proteins. The remaining ~ 10% consists of non-collagenous proteins that form the bone ground substance and play critical roles in bone formation, remodeling, and repair (Grabowski 2015).

Bone cells account for approximately 10% of the organic volume of bone tissue and are classified according to their lineage and function. Osteoprogenitor cells, which originate from mesenchymal stem cells (MSCs), differentiate into osteoblasts and subsequently osteocytes during bone maturation (Trompet et al. 2024). In contrast, osteoclasts arise from the hematopoietic lineage (Schwartz et al. 1976; Xing et al. 2005; Krishnan et al. 2006; Marx 2007; Bonewald and Johnson 2008; Chau et al. 2009; Schaffler and Kennedy 2012; Goldring 2015). Osteoprogenitor cells are distributed throughout the periosteum, endosteum, bone canals, and bone marrow, serving as a source of osteoblast precursors during bone formation and remodeling. Osteoblasts are the primary bone-forming cells responsible for extracellular matrix (ECM) synthesis and mineralization, a process mediated in part by the secretion of matrix vesicles (MVs). Beyond their bone-forming role, osteoblasts regulate osteoclastogenesis through the secretion of osteoprotegerin (OPG), a decoy receptor that inhibits RANKL-mediated osteoclast differentiation (Boyle et al. 2003).

Bone formation and maintenance depend on the migration, proliferation, and differentiation of MSCs into the osteoblastic lineage (Vidal et al. 1998; Yang and Karsenty 2002; Harada and Rodan 2003). This process is controlled by osteogenic transcription factors, particularly Runx2 and Osterix, which coordinate osteoblast commitment, differentiation, and maturation (Chau et al. 2009). Osteoblast activity is further regulated by hormones, cytokines, growth factors, and mechanical stimuli through signaling pathways such as BMP, Wnt/β-catenin, and MAPK, thereby coordinating bone formation and remodeling (Logan and Nusse 2004; ten Dijke and Hill 2004; Moon et al. 2004; Kolch 2005; Krishnan et al. 2006; Murphy and Blenis 2006; McKay and Morrison 2007; Anjum and Blenis 2008; Benitez et al. 2009; Derynck and Budi 2019; Carrillo-López et al. 2021; Chen et al. 2021).

## Biogenesis, molecular composition, and functional organization of MVs

MVs are mineralization-competent extracellular vesicles (EVs) ranging from 100 to 300 nm in diameter that are closely associated with the collagen-rich ECM, where they act as nanoreactors for biological apatite nucleation and propagation during biomineralization (Bolean et al. 2020). High-resolution microscopy studies have significantly advanced the understanding of MV biogenesis, supporting the concept that these vesicles predominantly originate through plasma membrane budding, particularly from microvillar regions, before being released into the ECM by osteoblasts, chondrocytes, odontoblasts, and cementoblasts (Rabinovitch and Anderson 1976; Borg et al. 1981; Akisaka and Shigenaga 1983; Hale and Wuthier 1987; Anderson et al. 2005; Julien et al. 2007; Garcés-Ortíz et al. 2013; Hayann et al. 2024, 2025, 2026). Although accumulating evidence supports the involvement of specialized membrane microdomains and endosomal pathways in MV formation (van Niel et al. 2018; Hayann et al. 2026), the precise mechanisms governing their biogenesis remain incompletely understood. The mineralization machinery associated with these vesicles includes tissue-nonspecific alkaline phosphatase (TNAP), nucleotide pyrophosphatase/phosphodiesterase 1 (NPP1), and PHOSPHO1, enzymes that coordinate phosphate metabolism and mineral ion availability during biomineralization. Together, they promote the accumulation of calcium and inorganic phosphate (Pi) within and around MVs, increasing local supersaturation and reducing the Gibbs free-energy barrier for biological apatite formation (Strates and Neuman 1958; Fleish and Neuman 1961; Kirsch et al. 1997; Sikirić and Füredi-Milhofer 2006; Chavkin et al. 2015; Yadav et al. 2016; Andrilli et al. 2023). TNAP hydrolyzes pyrophosphate (PPi) to generate Pi, while NPP1 regulates the local Pi/PPi ratio. Together, these enzymes control the relative abundance of Pi, a promoter of mineralization, and PPi, a potent inhibitor of biological apatite formation (Garimella et al. 2006; Bolean et al. 2020).

The mineralization capacity of MVs depends not only on their mineralization-related protein cargo but also on the specialized lipid environment of their membranes, which influences the localization and activity of mineralization-related proteins. (Camolezi et al. 2002; Ierardi et al. 2002; Bolean et al. 2010, 2011; Simão et al. 2013; Favarin et al. 2020, 2025). Lipids play a fundamental role in MVs structure and function, particularly those associated with lipid raft microdomains. MVs exhibit a lipid composition similar to that of parental cell lipid rafts, being enriched in cholesterol, sphingomyelin (SM), and ceramide (Cer) (Hayann et al. 2025). Recent lipidomic analyses identified more than 30 lipid species in MVs and demonstrated that changes in ionic conditions can alter both vesicular lipid composition and mineralization competence (Hayann et al. 2026).

Ceramide and SM have emerged as important regulators of biomineralization. Ceramide contributes to lipid raft stability and membrane remodeling, while SM participates in several bone-related cellular processes, including bone formation and mineralization (Gault et al. 2010; Airola and Hannun 2013). In mineralizing tissues, SM is hydrolyzed by sphingomyelin phosphodiesterase 3 (SMPD3) into ceramide and phosphocholine (PC). PC subsequently serves as a substrate for PHOSPHO1, contributing to intravesicular Pi production required for mineral formation, while SM-rich membrane domains have also been implicated in TNAP recruitment and anchoring (Hayann et al. 2026). Beyond their structural and mineralization-related functions, sphingolipids also act as important signaling molecules. Reduced levels of SM and ceramide have been associated with osteoporosis in mice (During et al. 2020). In addition, ceramide and its metabolites, including sphingosine, ceramide-1-phosphate, and sphingosine-1-phosphate, regulate cellular processes such as proliferation, apoptosis, migration, and adhesion (Airola and Hannun 2013).

Phosphatidylserine (PS), a negatively charged glycerophospholipid enriched in the inner leaflet of MVs membranes, is a key component of the nucleation core (Cotmore et al. 1971; McLean et al. 1987). PS-rich nanodomains promote the local accumulation of Ca^2+^ e Pi ions, leading to the formation and stabilization of PS-CaPi complexes, that serve as nucleation sites for mineral deposition (Wu et al. 1997; Cruz et al. 2020). Consistent with this model, infrared absorption spectroscopy and ^31^P-NMR analyses have identified that amorphous calcium phosphate (ACP), or its precursors, is the predominant mineral phase within the nucleation core (Wu et al. 1997; Favarin et al. 2025).

## Biomimetic models

**Fig. 1:**
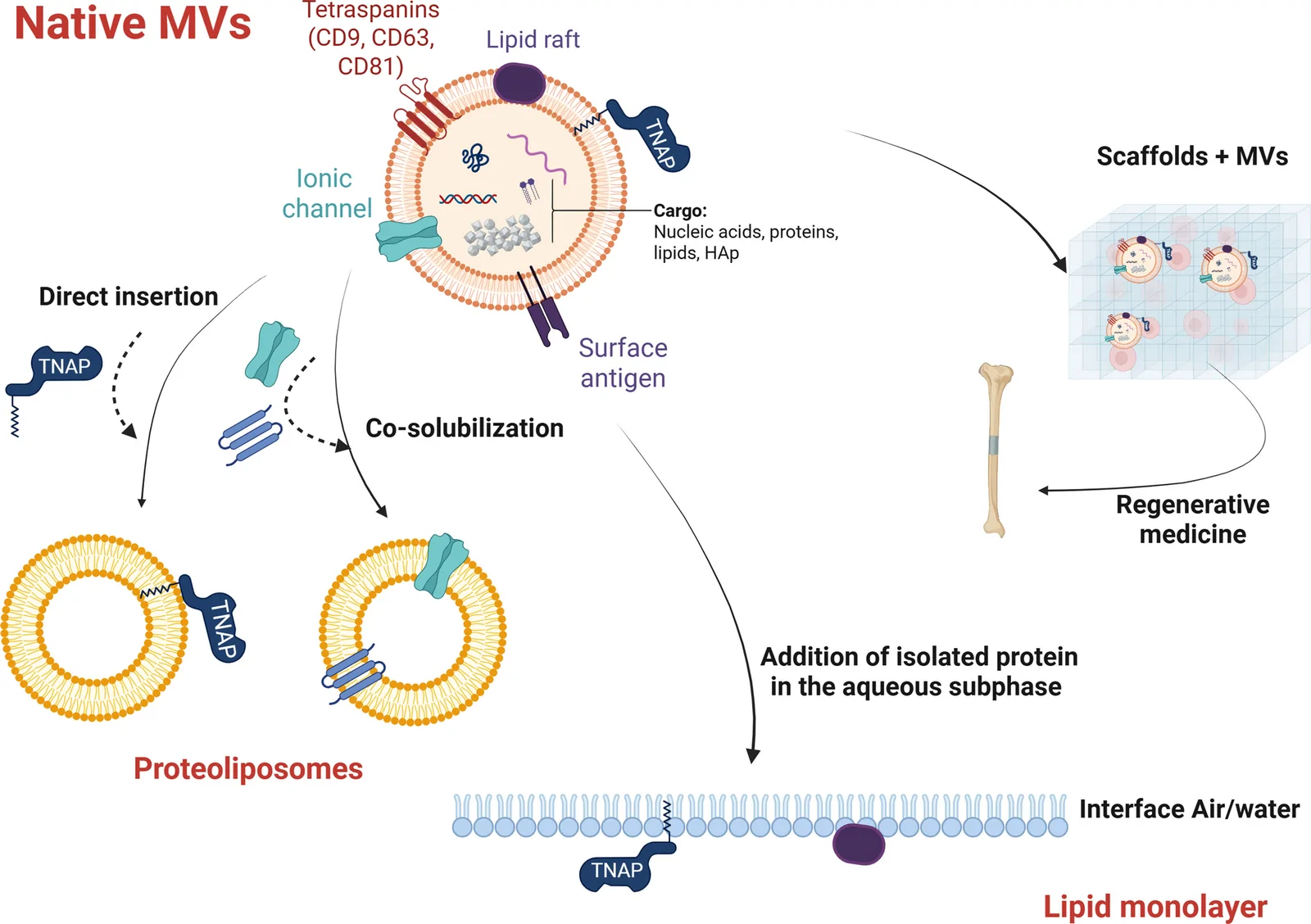
Native matrix vesicles (MVs) and biomimetic membrane models used to investigate osteogenic protein–lipid interactions. Matrix vesicles contain membrane proteins, including tissue-nonspecific alkaline phosphatase (TNAP), ion channels, tetraspanins (CD9, CD63, and CD81), surface antigens, and intravesicular cargo such as proteins, lipids, nucleic acids, and mineral precursors. A variety of biomimetic membrane systems have been developed. Depending on the biochemical and structural characteristics of the target protein, proteoliposomes can be generated through co-solubilization or direct insertion approaches. Alternatively, proteins may be incorporated into lipid monolayers. MVs can also be combined with three-dimensional extracellular matrix (ECM)-mimetic scaffolds to evaluate vesicle adhesion, retention, mineralization potential, and applications in bone tissue engineering and regenerative medicine

The structural, molecular, and functional complexity of MVs poses significant challenges to elucidating the individual contributions of their constituents to biomineralization. Although advances in biochemical, biophysical, and imaging techniques have greatly expanded our understanding of MV biology, dissecting the specific roles of proteins, lipids, and mineral phases within these highly dynamic systems remains difficult. To address this limitation, simplified biomimetic models have been developed to partially reproduce the composition, organization, and physicochemical properties of MVs, enabling the investigation of specific molecular mechanisms under controlled experimental conditions (Fig. 1).

### Liposomes and proteoliposomes

Membrane lipids, which are intrinsically insoluble in aqueous media in their monomeric form, spontaneously self-organize to form multilamellar particles in aqueous environments. Extrusion of multilamellar vesicles generates unilamellar vesicles, known as liposomes, which are composed of a single lipid bilayer closely resembling the architecture of biological membranes and typically exhibiting nanometric dimensions. These methodologies have been well-detailed and discussed in previous reviews (Ciancaglini et al. 2012; Ramos et al. 2017). Liposomal systems can be engineered to incorporate specific proteins and enzymes, generating proteoliposomes and enabling the reconstitution of their biological activity (Ciancaglini et al. 2012; Bolean et al. 2017). By reproducing selected structural and functional characteristics of MVs, proteoliposomes provide a highly controlled biomimetic model for studying the molecular mechanisms underlying MV-mediated biomineralization and calcification (Bolean et al. 2017; Bottini et al. 2018; Ramos et al. 2021).

The methodologies available for generating proteoliposomes depend on the type of interaction the protein has with the lipid membrane. Thus, if the goal is to study a peripheral protein, it can be encapsulated within the liposome, or its adsorption onto a pre-formed liposome can be investigated. Conversely, if the protein is an integral membrane protein, a solubilization and purification process is required first, followed by detergent-assisted reconstitution. For proteins featuring glycosylphosphatidylinositol (GPI) anchors, the most recommended strategy is the direct insertion of the protein into pre-assembled liposomes. This method is the most widely used in biomineralization studies (Ciancaglini et al. 1992; Ramos et al. 2021).

Biomimetic studies using TNAP-proteoliposomes have provided important insights into the mechanisms by which membrane organization and lipid composition regulate enzyme activity and mineral propagation (Camolezi et al. 2002; Bolean et al. 2010; Garcia et al. 2015; Favarin et al. 2017, 2025). Purified TNAP can be reconstituted into liposomal membranes through its GPI anchor, generating proteoliposomes with sizes ranging from 100 to 200 nm that retain their catalytic activity toward a broad spectrum of substrates, including ATP, ADP, AMP, PPi, and p-nitrophenyl phosphate (pNPP) (Camolezi et al. 2002; Bolean et al. 2010; Favarin et al. 2017, 2025). Notably, TNAP-proteoliposomes are capable of inducing mineral formation even in the absence of additional nucleators, emphasizing the contribution of the membrane composition itself to mineral nucleation and propagation. Variations in phospholipid and sterol content also affect mineral deposition efficiency, underscoring the importance of membrane organization and fluidity during biomineralization (Favarin et al. 2017, 2020; Andrade et al. 2019).

In addition to TNAP, proteoliposome-based models have been employed to investigate other MV-associated proteins, including NPP1, Na^+^/K^+^-ATPase, and annexins (Sebinelli et al. 2019; Veschi et al. 2020; Andrilli et al. 2023, 2024). These biomimetic systems have also proven valuable for studying the cooperative and competitive interactions among enzymes involved in phosphate metabolism (Amadeu de Oliveira et al. 2024), as well as protein–protein interactions relevant to biomineralization (Veschi et al. 2020; Pasquarelli et al. 2022). Furthermore, proteoliposomes have been used to evaluate the effects of disease-associated mutations and the screening of compounds capable of modulating enzyme activity (Di Mauro et al. 2002; Lanier et al. 2010; Rauner et al. 2017; Itel et al. 2018; Wei et al. 2020). Collectively, these findings highlight the versatility of proteoliposomes as experimental platforms for dissecting the molecular mechanisms governing both physiological and pathological mineralization.

### Giant unilamellar vesicles (GUVs)

Giant unilamellar vesicles (GUVs) are valuable biomimetic membrane models for studying molecular processes involved in MV biology and biomineralization. Their cell-sized dimensions enable direct visualization of membrane architecture, lipid phase behavior, membrane dynamics, and protein–membrane interactions, providing complementary insights into membrane organization that are difficult to obtain with nanoscale vesicle models (Kahya 2010; Litschel and Schwille 2021). A recent review has extensively described this technique (Fletcher et al. 2025).

Studies using GUVs composed of 1,2-dioleoyl-sn-glycero-3-phosphocoline (DOPC) or a mixture of DOPC and 1,2-dipalmitoyl-sn-glycero-3-phospho-L-serine (DPPS) demonstrated that annexin A5 functionally interacts with model membranes and mediates Ca^2+^ transport across lipid bilayers, providing mechanistic insights into its proposed role during MV-mediated mineralization (Bolean et al. 2015). In another study, McDonough et al (2024) developed GUVs that mimic the lipid asymmetry of the plasma membrane by enriching phosphatidylserine in the inner leaflet. This biomimetic model provides a more physiologically relevant platform for studying lipid–protein interactions and membrane organization, highlighting the potential of engineered GUVs to investigate the molecular mechanisms underlying matrix vesicle function and biomineralization (McDonough et al. 2024).

Owing to their micrometer-sized dimensions, GUVs are particularly advantageous for confocal microscopy studies, enabling the visualization of lipid domain formation and dynamic membrane processes under controlled conditions (Chan and Boxer 2007). Combined with fluorescent-based approaches such as fluorescent recovery after photobleaching (FRAP), these systems allow quantitative evaluation of membrane fluidity, molecular diffusion, and lateral organization. Together, these methodologies provide valuable mechanistic insights into the dynamic protein-lipidic interactions that regulate MVs’ functions during biomineralization (Lippincott-Schwartz et al. 2018).

### Langmuir monolayer and Langmuir–Blodgett (LB)

Langmuir monolayer and Langmuir–Blodgett (LB) films constitute a valuable biomimetic interfacial model for investigating leaflet membrane-associated processes relevant to MVs biology. These systems mimic a single membrane leaflet and provide a versatile platform for studying phase behavior, molecular packing, and interactions with solutes or macromolecules present in an aqueous subphase. Unlike vesicular systems, which reproduce closed membrane structures, Langmuir monolayers offer a simplified two-dimensional platform that enables precise control of lipid packing, surface pressure, and molecular organization at the air–water interface. They are formed by spreading amphiphilic molecules onto an aqueous subphase, where the polar headgroups remain in contact with the aqueous environment while the hydrophobic chains orient toward the air, thereby reproducing the organization of a membrane leaflet (Caseli et al. 2015; Ramos et al. 2021; Oliveira et al. 2022). The composition of the subphase can be readily adjusted to reproduce specific physicochemical conditions, making these systems particularly useful for investigating lipid–lipid, lipid–protein, and lipid–mineral interactions.

Compression of Langmuir monolayers generates surface pressure–area (π–A) isotherms that provide quantitative information on molecular packing, miscibility, phase behavior, compressibility, and intermolecular interactions. Furthermore, transfer of monolayers onto solid substrates produces LB films, enabling complementary structural and functional analyses (Caseli et al. 2015). These approaches have been extensively employed to investigate the interactions of MV-associated proteins with lipid membranes. Studies using monolayers composed of DPPC and cholesterol demonstrated that lipid domains modulate TNAP insertion, distribution, and catalytic activity, highlighting the influence of membrane organization on enzyme function (Andrade et al. 2019; Derradi et al. 2019).

Langmuir monolayers have also proven useful for investigating the role of phosphatidylserine-rich microenvironments during biomineralization. Monolayers containing DPPS exhibited increased stability in the presence of Ca^2+^ and annexin A5, supporting the concept that these components cooperate to stabilize membrane domains involved in mineral formation (Ferreira et al. 2023). Moreover, DPPS-containing monolayers could induce mineral nucleation under biomimetic conditions, reproducing key features of the nucleation process observed in native MVs (Cruz et al. 2020). Similar findings were obtained using lipids extracted from native MVs, further supporting the central role of PS in the initiation of biomineralization.

Beyond fundamental studies of membrane organization, Langmuir monolayers and LB films have also enabled the development of functional biomimetic materials. For instance, TNAP-containing LB films transferred onto titanium substrates retained enzymatic activity and promoted apatite deposition, demonstrating their potential as bioinspired coatings for mineralization-inducing surfaces (Andrade et al. 2019). Collectively, these studies highlight the versatility of Langmuir-based models for elucidating how membrane composition, protein adsorption, and interfacial physicochemical properties regulate MVs’ function and biomineralization.

## Experimental approaches for investigating osteogenic proteins at lipid interfaces

The development of biomimetic membrane systems has significantly advanced the investigation of osteogenic proteins associated with MVs (Bottini et al. 2018). Nevertheless, a comprehensive understanding of the molecular mechanisms governing biomineralization requires the characterization of both native EVs, including matrix MVs and media vesicles (also called exosomes), and simplified biomimetic membrane models designed to reproduce specific structural and functional features of these systems (Bottini et al. 2018). Over the past decades, a wide range of biophysical, imaging, and spectroscopic techniques has been employed to investigate lipid interfaces across these complementary experimental platforms. Together, these methodologies provide multiscale information spanning membrane organization, protein–lipid interactions, vesicle heterogeneity, mineral nucleation, and calcium phosphate phase evolution, offering valuable insights into the mechanisms underlying physiological and pathological biomineralization.

**Fig. 2:**
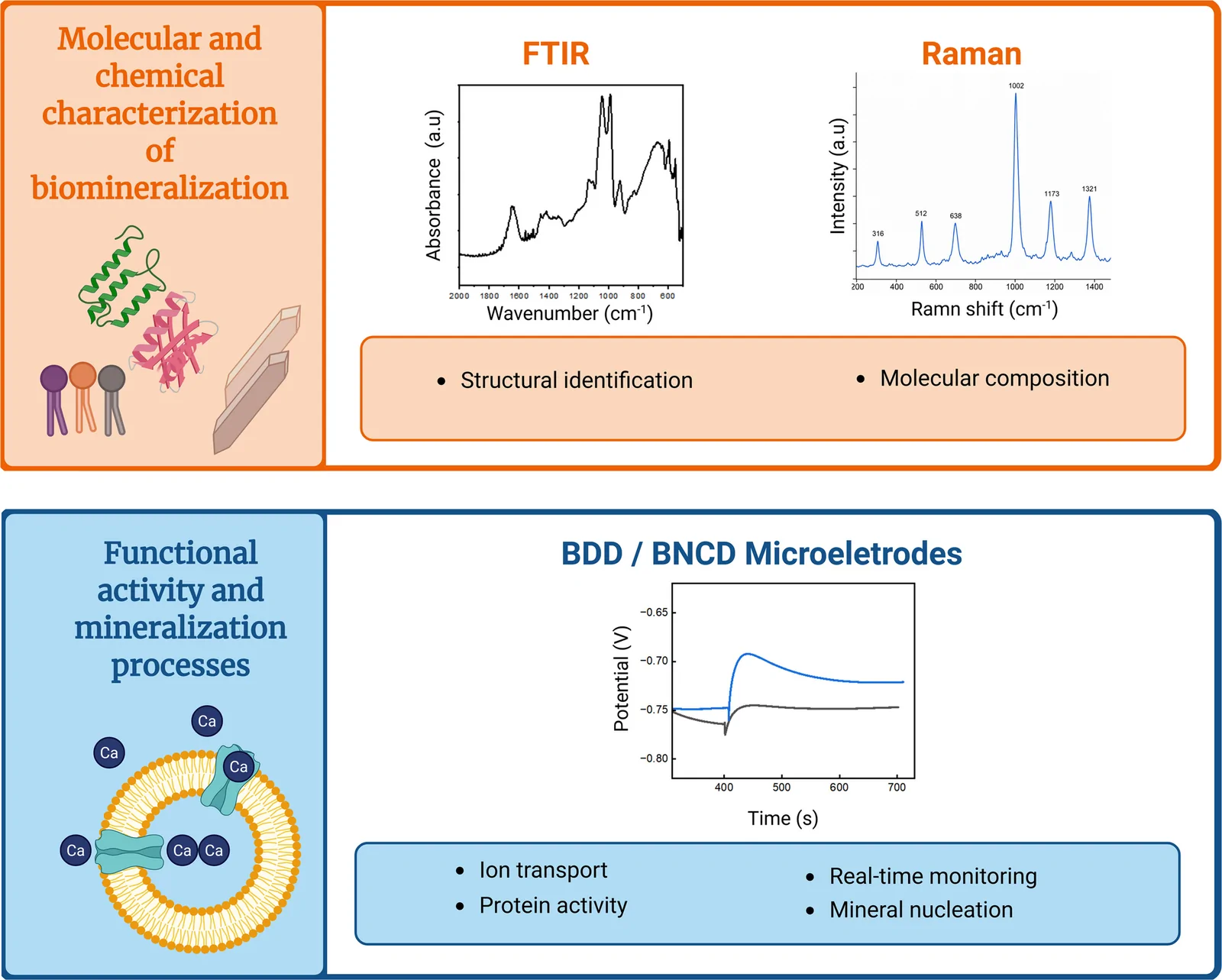

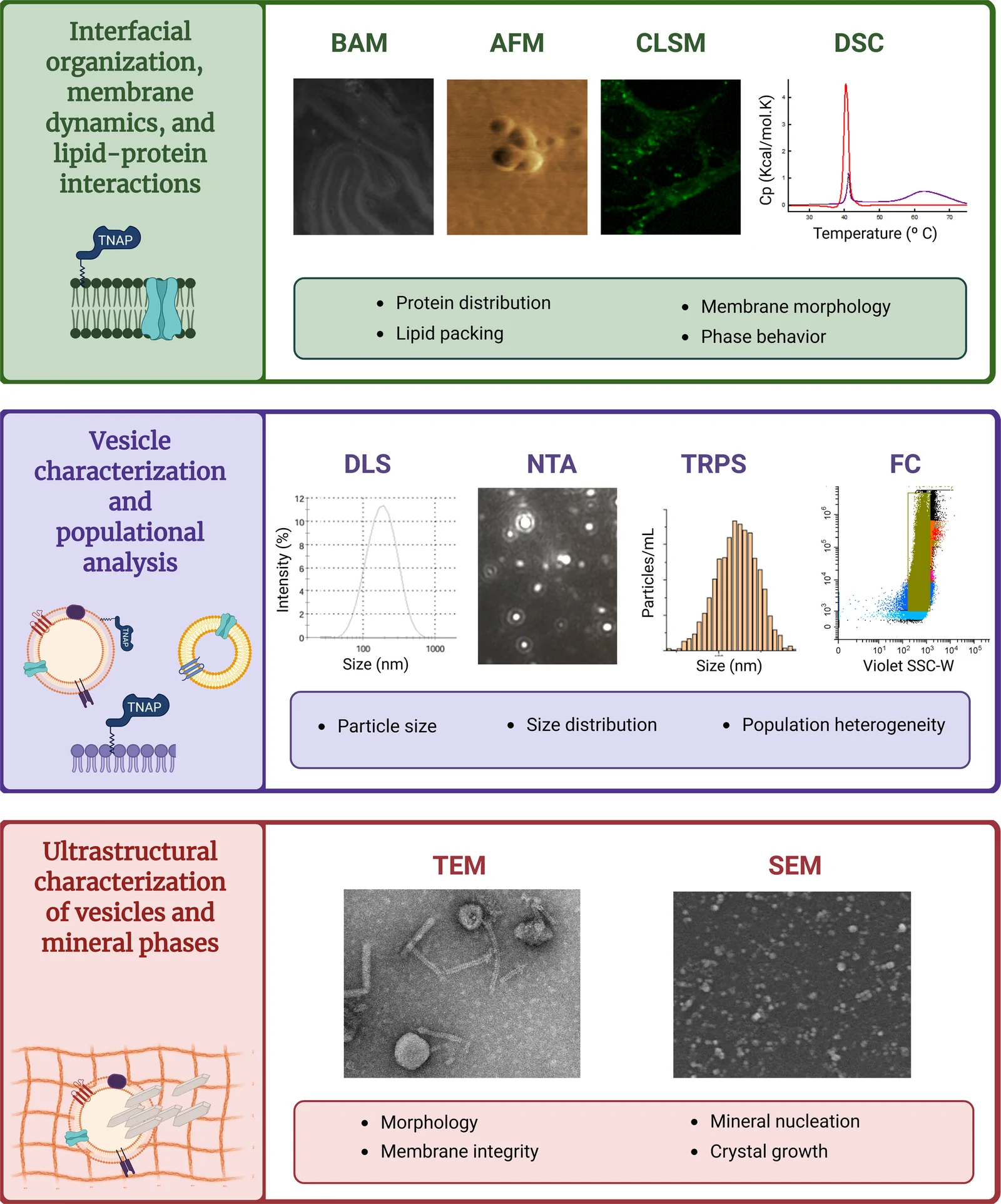
Multiscale analytical toolbox for investigating extracellular vesicles (EVs), matrix vesicles (MVs), and biomineralization processes. Top panel: Molecular and chemical characterization of mineral phases using Fourier-transform infrared spectroscopy (FTIR) and Raman spectroscopy, providing information on molecular composition, crystallinity, and structural signatures of biominerals. Functional activity associated with mineralization is monitored using boron-doped diamond (BDD) and boron/nitrogen co-doped diamond (BNCD) microelectrodes, enabling real-time measurements of ion transport, protein activity, and mineral nucleation events. Bottom panel: Interfacial organization and membrane properties are assessed through Brewster angle microscopy (BAM), atomic force microscopy (AFM), confocal laser scanning microscopy (CLSM), and differential scanning calorimetry (DSC), revealing lipid packing, protein distribution, membrane morphology, and phase behavior. Vesicle populations can be characterized by dynamic light scattering (DLS), nanoparticle tracking analysis (NTA), tunable resistive pulse sensing (TRPS), and flow cytometry (FC), providing complementary information on particle size, size distribution, concentration, and population heterogeneity. Ultrastructural characterization is performed using transmission electron microscopy (TEM) and scanning electron microscopy (SEM), allowing visualization of vesicle morphology, membrane integrity, mineral nucleation, and crystal growth. Together, these complementary methodologies provide a comprehensive framework for studying the physicochemical properties, biological activity, membrane organization, and mineralization capacity of EVs and MVs in biomineralization systems

As illustrated in Fig. 2, these methodologies provide information at different levels of biological and physicochemical organization. A summary of the principal techniques discussed throughout this review, together with their main applications and associated model systems, is presented in Table 1.

**Table 1:** Summary of analytical techniques used for investigating osteogenic protein in lipid interfaces

Technique	Typical samples	Main information obtained	Applications in biomineralization studies	Main limitations
BAM	Langmuir monolayers	Lipid domains, phase separation, protein-induced membrane reorganization	TNAP partitioning, mineral precursor formation, membrane heterogeneity	Limited to mesoscale resolution
AFM	MVs, EVs, proteoliposomes, supported lipid bilayers	Topography, roughness, elasticity, adhesion forces, Young's modulus	Vesicle morphology, membrane remodeling, mineral nucleation, nanomechanics	Surface analysis only
CLSM	Fluorescently labeled MVs, EVs, GUVs, cells, ECM scaffolds	Protein localization, lipid distribution, colocalization, vesicle uptake	Tracking osteogenic proteins, membrane heterogeneity, vesicle–ECM interactions	Requires fluorescent labeling
DSC	Liposomes, proteoliposomes, lipid mixtures, proteins	Phase transitions, Tm, ΔH, ΔS, membrane cooperativity	TNAP incorporation, lipid domain formation, membrane stability	Indirect structural information
DLS	MVs, EVs, liposomes, proteoliposomes	Hydrodynamic diameter, PDI, colloidal stability	Vesicle characterization, quality control, aggregation studies	Biased toward larger particles
NTA	MVs, EVs, liposomes, proteoliposomes	Particle size, concentration, population heterogeneity	Quantification of vesicle populations and subpopulations	Limited detection of very small particles
TRPS	MVs, EVs, liposomes, proteoliposomes	Size, concentration, zeta potential (single-particle)	Population heterogeneity and surface charge analysis	Requires nanopore calibration
FC	MVs, EVs, biological fluids	Surface markers, phenotypic profiling, subpopulation analysis	Detection of TNAP +, LC3 +, and osteogenic vesicle subsets	Limited sensitivity for very small vesicles
TEM	MVs, EVs, proteoliposomes, mineral phases	Ultrastructure, morphology, mineral deposits	Mineral nucleation, vesicle morphology, crystal identification (SAED)	Sample preparation artifacts
SEM	MVs, EVs, scaffolds, mineralized matrices	Surface morphology and mineral deposition	Vesicle–collagen interactions, mineral propagation, scaffold colonization	Limited internal structural information
FTIR	Liposomes, proteoliposomes, MVs, mineral phases	Protein secondary structure, lipid order, mineral composition	Protein–membrane interactions, ACP/OCP/HAp formation	Spectral overlap can complicate interpretation
Raman	Liposomes, proteoliposomes, MVs, mineral phases	Molecular composition, lipid order, mineral phase identification	Monitoring mineral maturation and membrane organization	Weak signals without enhancement strategies
BDD/BNCD	Proteoliposomes, membrane-mimetic systems	Real-time ion transport and electrochemical changes	Monitoring Ca^2^⁺ transport and membrane-associated mineralization events	Limited number of biomineralization applications

*AFM* atomic force microscopy, *BAM* Brewster angle microscopy, *BDD* boron-doped diamond, *BNCD* boron-doped nanocrystalline diamond, *CLSM* confocal laser scanning microscopy, *DLS* dynamic light scattering, *DSC* differential scanning calorimetry, *FC* flow cytometry, *FTIR* Fourier-transform infrared, *IFC* imaging flow cytometry, *NTA* nanoparticle tracking analysis, *SEM* scanning electron microscopy, *TEM* transmission electron microscopy, *TRPS* tunable resistive pulse sensing, *ACP* amorphous calcium phosphate, *OCP* octacalcium phosphate, *HAp* hydroxyapatite

### Interfacial organization, membrane dynamics, and lipid-protein interactions

Characterizing the organization and properties of lipid interfaces is fundamental for understanding how osteogenic proteins regulate biomineralization in both native EVs and biomimetic membrane systems. A range of complementary techniques has therefore been employed to investigate membrane morphology, lipid packing, phase behavior, and protein distribution at these interfaces, providing detailed insights into membrane organization, physicochemical properties, and lipid–protein interactions that govern vesicle function.

#### Brewster angle microscopy (BAM)

BAM is a label-free imaging technique employed in biomineralization studies using Langmuir monolayer models. The technique is based on Brewster’s Law, which describes the angle at which *parallel-*polarized light (p-polarized light) is not reflected at the interface between media with different refractive indices (Hénon and Meunier 1991; Hoenig and Moebius 1991).

Owing to its mesoscale resolution (typically 10–100 µm), corresponding to the characteristic size range of many lipid domains, BAM is particularly useful for monitoring lipid phase separation, domain formation and morphology, membrane heterogeneity, and protein-induced structural rearrangements that cannot be obtained from surface pressure-area isotherms alone (Prenner et al. 2007; Gröger et al. 2012). In the context of MV-mediated biomineralization, BAM was used to investigate TNAP interactions with DPPC/cholesterol Langmuir monolayers, revealing a preferential association of the enzyme with cholesterol-rich membrane domains and demonstrating the influence of membrane organization on protein incorporation and activity (Derradi et al. 2019). Beyond studies on MVs, BAM has also been applied to monitor mineral nucleation processes in real-time. Coupling BAM to a Langmuir trough enabled the visualization of amorphous calcium carbonate (ACC) precursor formation and phase transformations beneath phospholipid monolayers (Xiao et al. 2010), while ACC nucleation and growth during biomimetic CaCO_3_ mineralization were observed in stearic acid monolayers (Chen et al. 2009). These applications demonstrate the ability of BAM to directly monitor dynamic structural changes associated with mineral formation.

#### Atomic force microscopy (AFM)

AFM is a powerful nanoscale imaging technique widely used to investigate membranes in biomimetic systems, MVs, and other types of EVs. Unlike electron microscopy, AFM does not require conductive samples or extensive preparation, enabling high-resolution characterization of membrane-associated structures in air or liquid environments (García 2002; Müller and Dufrêne 2008; Dufrêne et al. 2017). In addition, the technique enables the characterization of EV heterogeneity at the single-vesicle level, revealing structural features often obscured in light scattering measurements. Despite its versatility, AFM is limited to surface characterization and may be affected by probe geometry or sample deformation during scanning. Consequently, it is often integrated with complementary microscopy or spectrometry approaches, enabling the simultaneous correlation of multiple structural, mechanical, and physicochemical parameters and providing a more comprehensive characterization of biological and biomimetic membrane systems (Khanal et al. 2020; Bagrov et al. 2023; Buccini et al. 2024).

AFM provides quantitative information on membrane roughness, vesicle morphology, elasticity, nanoscale organization and properties, including adhesion forces, membrane stiffness, and Young’s modulus. In biomineralization studies, the technique has been particularly valuable for visualizing protein-induced membrane remodeling events, such as lipid domain formation, protein clustering, and membrane reorganization associated with mineral nucleation. Together, these structural and mechanical measurements contribute to the understanding of osteogenic protein-lipid interactions and membrane behavior during biomineralization (Uchihashi et al. 2012; Alsteens et al. 2017; Sebinelli et al. 2019).

#### Confocal laser scanning microscopy (CLSM)

CLSM is a fluorescence-based imaging technique that enables optical sectioning and three-dimensional reconstruction of biological structures under near-physiological conditions. Owing to its ability to selectively visualize fluorescent-labeled molecules, CLSM has become an important tool for investigating EVs and biomimetic membrane systems. Although its spatial resolution is limited by optical diffraction resolution and relies on fluorescent labeling, CLSM provides unique molecular information that cannot be readily obtained using purely structural imaging techniques (Théry et al. 2018).

In studies of biomineralization, CLSM is particularly valuable because it enables the direct identification and spatial localization of osteogenic proteins, membrane lipids, and ECM components using fluorescent dyes, labeled probes, and antibody-conjugated fluorophores. This molecular specificity distinguishes CLSM from techniques such as BAM and AFM, which provide detailed structural and biophysical information but cannot generally directly identify individual molecular species.

Consequently, CLSM enables the investigation of protein localization, membrane heterogeneity, lipid domain formation, and dynamic membrane reorganization (Bagatolli and Gratton 1999; Bolean et al. 2010, 2017; Dimova 2019). When combined with fluorescence recovery after photobleaching (FRAP), CLSM can additionally provide quantitative information on the mobility and lateral diffusion of fluorescently labeled membrane components, allowing aspects of membrane dynamics to be investigated. FRAP measures the recovery of fluorescence within a photobleached region, providing information on molecular mobility and the fraction of mobile and immobile components within the membrane (Epand et al. 2009; Day and Kang 2023).

The technique also supports co-localization analyses, allowing the investigation of spatial relationships between proteins, lipids, and specific membrane domains involved in mineralization. Such analyses are important for determining whether osteogenic proteins preferentially associate with lipid microenvironments that regulate membrane organization and mineralization activity. Furthermore, time-resolved CLSM imaging, particularly when combined with approaches such as FRAP, can be used to monitor dynamic events such as vesicle trafficking, protein recruitment, and membrane remodeling in real-time (Bagatolli et al. 2003; Simons and Vaz 2004).

#### Differential scanning calorimetry (DSC)

DSC is a thermoanalytical technique used to investigate the thermodynamic behavior of proteins, lipids, and biomembrane systems. The method measures changes in heat capacity as a function of temperature, enabling the characterization of phase transitions, protein stability, lipid-protein interactions, lipid packing and the effects of protein incorporation on membrane structure (Spink 2008; Durowoju et al. 2017). Although DSC does not provide direct structural visualization, it offers unique quantitative information on membrane thermodynamics that complements imaging and spectroscopic techniques.

DSC has been extensively employed to characterize MV-inspired proteoliposomes. The technique has proven particularly useful for evaluating the reciprocal effects of membrane composition on protein function and of proteins on membrane organization. Calorimetric analysis revealed that cholesterol-containing vesicles promote lateral phase segregation and microdomain formation, while the incorporation of TNAP modifies membrane thermotropic behavior and lipid phase transition temperatures (Bolean et al. 2010, 2011; Favarin et al. 2017, 2020). In addition, the degree of TNAP incorporation into proteoliposomes is dependent on membrane lipid composition (Simão et al. 2019).

The applicability of DSC extends beyond membrane systems and protein-lipid interactions. The technique has been employed to investigate ECM components involved in biomineralization, particularly collagen, which serves as a major scaffold for mineral deposition. Changes in this protein thermal behavior have been explored as potential biomarkers for osteosarcoma detection, as tumor progression alters collagen degradation patterns and leads to the formation of more thermally stable protein fragments (Lamonaca et al. 2016). Collectively, DSC remains an important component of the biophysical set of techniques used to investigate both physiological and pathological biomineralization.

### Vesicles characterization and populational analysis

Characterization of vesicle populations is essential for validating biomimetic models and understanding the physicochemical properties of both native and synthetic vesicles. Parameters such as particle size, size distribution, concentration, and population heterogeneity directly influence vesicle behavior and may affect the interpretation of functional assays. Several complementary techniques have been employed to characterize vesicle populations and evaluate the effects of protein incorporation, lipid composition, and mineralization conditions on vesicle properties.

#### Dynamic light scattering (DLS)

DLS is a non-destructive technique frequently used for the characterization of liposomes, EVs, and nanoparticles owing to its low sample requirements, rapid analysis, and compatibility with diverse experimental conditions. The technique estimates the hydrodynamic diameter and size distribution from the fluctuations in scattered light generated by Brownian motion (Stetefeld et al. 2016).

DLS has been employed in several biomineralization studies to determine vesicle size distribution, colloidal stability, and aggregation behavior. Variations in particle size and polydispersity index (PDI) provide valuable information regarding membrane heterogeneity, vesicle fusion, clustering, and mineral nucleation processes (Bolean et al. 2010; Garcia et al. 2015; Melo et al. 2026). The technique is also useful for monitoring temporal changes in vesicle stability and protein-induced structural remodeling under different physicochemical conditions (Andrilli et al. 2023). Furthermore, DLS can be employed as a quality-control approach to evaluate the reproducibility of vesicle preparations. Batch-to-batch comparisons of hydrodynamic diameter and PDI provide a rapid assessment of population homogeneity and preparation consistency, which is essential for ensuring the reliability of subsequent functional and biomineralization assays.

However, because scattering intensity is strongly biased toward larger particles and aggregates, heterogeneous systems often require complementary techniques such as electron microscopy or cryogenic imaging for more detailed characterization (Gardiner et al. 2013; Bhattacharjee 2016; Théry et al. 2018). An alternative approach is size exclusion chromatography coupled with DLS (SEC-DLS), in which chromatographic separation prior to DLS detection can help reduce the influence of larger particles and aggregates on the measured size distribution, enabling the characterization of distinct particle populations according to their elution profiles. SEC-DLS can therefore provide complementary information for heterogeneous EV preparations and improve the interpretation of DLS measurements (Varga et al. 2014).

#### Nanoparticle tracking analysis (NTA)

NTA enables the simultaneous determination of particle size and concentration, making it particularly suitable for the characterization of MVs and biomimetic membrane systems. Diffusion coefficients obtained from single-particle tracking of Brownian motion are converted into hydrodynamic diameters using the Stokes–Einstein relationship. The ability to quantify particle concentration and size distribution enables the detection of population shifts that may not be apparent using DLS, while also allowing individual particle analysis (Dragovic et al. 2011; Gardiner et al. 2013; Mehdiani et al. 2015). Modern NTA platforms operate in both light-scattering and fluorescence modes. While scattering mode provides measurements of particle size distribution and concentration, fluorescence-NTA allows selective detection of labeled vesicle subpopulations (Mehdiani et al. 2015; Théry et al. 2018).

In biomineralization studies, NTA have been particularly useful for the determination of particle concentration and investigating changes in vesicle populations associated with protein composition, membrane remodeling, and vesicle stability (Cominal et al. 2025; Hayann et al. 2026).

Despite its advantages, NTA has limitations, including reduced sensitivity for particles smaller than 50–70 nm and lower accuracy in highly concentrated or polydisperse samples. In addition, NTA cannot reliably distinguish vesicles from protein aggregates or mineral particles without fluorescence labeling (Gardiner et al. 2013; Mehdiani et al. 2015).

#### Tunable resistive pulse sensing (TRPS)

TRPS is an established single-particle analytical technique that characterizes vesicle populations through electrical detection rather than light scattering. Based on the Coulter principle (Willmott 2018), the method measures transient changes in ionic current generated as individual particles traverse a tunable nanopore and temporarily disrupt ion flow. The use of an adjustable elastomeric nanopore allows optimization of the measurement range for particles of different sizes, making TRPS suitable for heterogeneous vesicle populations (Weatherall and Willmott 2015; Mehdiani et al. 2015). Despite its advantages, TRPS requires careful nanopore calibration and appropriate matching between pore size and particle dimensions (Coumans et al. 2014).

Applications of TRPS in biomineralization remain relatively limited; however, the technique has been widely applied and validated for extracellular vesicle characterization and provides valuable single-particle measurements of size, concentration, and zeta potential (Coumans et al. 2014).

#### Flow cytometry (FC)

FC is a laser-based technique that characterizes individual particles in hydrodynamic flow based on their light-scattering and fluorescence properties (Shapiro 2003). Although conventional flow cytometers were originally developed for cellular analysis and have limited sensitivity for nanoscale particles, advances in optics, detectors, and instrument design have enabled dedicated nanoscale flow cytometers, significantly improving EV characterization. However, conventional instruments remain limited in detecting particles below 500 nm, where weak light-scattering signals, electronic noise, and swarm detection can compromise quantitative accuracy (Kim et al. 2024). Imaging flow cytometry (IFC) combines conventional FC with high-resolution imaging, enabling simultaneous fluorescence analysis and visualization of individual EVs. This allows assessment of vesicle morphology, colocalization, cellular uptake, and discrimination of artefacts such as protein aggregates, debris, and non-vesicular fluorescent particles (Ofir-Birin et al. 2018; Ricklefs et al. 2019).

In biomineralization research, FC-based approaches have been employed to characterize vesicle subpopulations, quantify membrane-associated biomarkers, and investigate molecular changes associated with osteogenic differentiation, bone regeneration, and pathological calcification. The detection of TNAP, LC3, and other osteogenic proteins was evaluated, as well as the monitoring of changes in vesicle populations under different experimental conditions (Li et al. 2022; Man et al. 2023). Welsh et al. (2023) provide a comprehensive framework for single-EV flow cytometry, covering instrument calibration, assay design, fluorescence controls, data acquisition, and standardized reporting to improve reproducibility across studies (Welsh et al. 2023).

### Ultrastructural characterization of vesicles and mineral phases

Direct visualization of vesicles and mineral structures provides critical information that cannot be obtained solely through bulk biophysical measurements. Electron microscopy techniques enable the examination of vesicle morphology, membrane integrity, mineral nucleation, and crystal growth with high spatial resolution, making them indispensable tools for investigating both native MVs and biomimetic systems. Furthermore, recent cryogenic approaches have expanded the ability to analyze hydrated samples while minimizing preparation-induced artifacts.

#### Transmission electron microscopy (TEM) and cryogenic transmission electron microscopy (cryo-TEM)

TEM is one of the principal high-resolution techniques used to investigate nanoscale membrane-associated mineralization systems. By transmitting an electron beam through ultrathin specimens, TEM enables detailed visualization of vesicle morphology, membrane organization, protein assemblies, and early mineral nucleation events.

The technique has been widely applied to characterize MVs and EVs, proteoliposomes, and other biomimetic membrane systems. In addition, selected-area electron diffraction (SAED) can be combined with a TEM platform to identify crystalline mineral phases and distinguish amorphous from crystalline calcium phosphate structures during mineral formation and propagation (Anderson 2003; Franken et al. 2006; Williams and Carter 2009; Golub 2011; Cui et al. 2016).

Despite its high spatial resolution, conventional TEM requires dehydration, staining, and high-vacuum conditions, which may alter the native organization of biological and synthetic membranes. Cryo-TEM overcomes many of these limitations by preserving samples in a vitrified hydrated state. This approach enables the visualization of membrane ultrastructure, vesicle architecture, and mineral phases with minimal sample perturbation, and has become particularly important for studying membrane remodeling, lipid domain formation, membrane curvature, and protein clustering associated with biomineralization processes (Dubochet et al. 1988; Kühlbrandt 2014; Beck and Baumeister 2016). In addition, analysis of TNAP-proteoliposomes by Cryo-TEM enabled the direct visualization of calcium phosphate precursors associated with PS-rich membranes, while proteoliposomes reconstituted with TNAP mutants failed to generate biological apatite-like structures (Favarin et al. 2025).

#### Scanning electron microscopy (SEM) and cryogenic scanning electron microscopy (Cryo-SEM)

SEM is a high-resolution imaging technique widely employed to investigate the surface morphology and three-dimensional organization of biological and mineralized systems. Unlike TEM, which reveals internal structure, SEM generates images of the specimen surface with a focused electron beam, enabling visualization of membrane topography, mineral deposits, and supramolecular organization (Anderson 2003; Goldstein et al. 2018).

SEM has been extensively used to characterize MVs and biomimetic membrane systems, providing information on vesicle morphology, mineral growth, membrane-associated aggregates, and spatial organization of mineralizing structures (Cavallaro et al. 2021; Iwayama et al. 2022; Favarin et al. 2020; Favarin et al. 2025; Ciancaglini et al., 2010). Furthermore, SEM analysis allows the investigation of the interactions between vesicles and ECM-inspired substrates. Recent studies demonstrated the adhesion and spatial distributions of MVs and media-EVs on three-dimensional collagen scaffolds, enabling the visualization of vesicle-matrix interactions, evaluation of binding potential, and mineral precursor deposition into ECM (Nogueira et al. 2025; Melo et al. 2026).

Conventional SEM requires fixation, dehydration, and conductive coating of biological samples, procedures that may introduce structural artifacts and alternative membrane organization. To minimize these limitations, approaches such as environmental SEM (ESEM), low-voltage SEM, and cryogenic SEM (cryo-SEM) have been developed to preserve hydrated specimens under conditions closer to their native state. In combination with energy-dispersive X-ray spectroscopy (EDS), SEM also enables simultaneous analysis of morphology and mineral composition, facilitating identification of calcium- and phosphate-rich mineral phases (Goldstein et al. 2018).

### Molecular and chemical characterization of biomineralization

While imaging techniques reveal structural aspects of vesicles and mineral deposits, molecular-level characterization is required to identify the chemical species involved in biomineralization and to monitor their transformations during mineral formation. Understanding how key bone-inducing proteins, such as bone morphogenetic proteins (BMPs) and TNAP, undergo conformational changes in their secondary structure upon interaction with lipid membranes or during mineral nucleation depends on the analysis of deconvolved vibrations of the polypeptide backbone (Mechiche Alami et al. 2017; Bispo et al. 2025). Vibrational spectroscopies have therefore become essential tools for this investigation.

#### Four-transform infrared (FTIR)

FTIR is a vibrational spectroscopic technique used to investigate protein conformation, lipid organization, and protein-membrane interactions in biological and biomimetic membrane systems. The technique measures molecular vibrations associated with changes in dipole moment, providing detailed information on the structural organization of both proteins and lipids (Petibois et al. 2010; Gieroba et al. 2021).

Fourier-transform infrared (FTIR) spectroscopy evaluates molecular vibrations associated with dipole moment changes, providing structural insights into protein conformation, lipid organization, and mineral phases (Petibois et al. 2010; Gieroba et al. 2021). In membrane-protein systems, the Amide I (~ 1600–1700 cm^−1^) and Amide II (~ 1545 cm^−1^) bands monitor conformational changes in polypeptide backbones upon membrane insertion. The hydrophobic insertion or anchoring of these enzymes in phospholipid bilayers alters the spectral contributions of α-helices (~ 1650–1655 cm^−1^) and β-sheets (~ 1620–1640 cm^−1^) (Ahiri 2026), reflecting allosteric adjustments that coordinate Ca^2+^ and P_*i*_ ions at the membrane interface (Nogueira et al. 2025; Bispo et al. 2025). Concurrently, symmetric and asymmetric CH_2_ stretching modes (~ 2850–2920 cm^−1^) track lipid acyl chain packing and phase transitions (L_β_ → L_α_).

In biomineralization studies, FTIR is pivotal for identifying mineral phases and tracking their structural evolution. The progressive emergence and splitting of phosphate (${PO}_{4}^{3-}$) vibrational modes specifically the ν_1_/ν_3_ stretching (~ 900–1200 cm^−1^) and ν_4_ bending (~ 560–600 cm^−1^) bands serve as spectral fingerprints to discriminate amorphous calcium phosphate (ACP) from octacalcium phosphate (OCP) and poorly crystalline, carbonate-substituted biological apatite (H_Ap_). Furthermore, FTIR enables quantitative assessment of mineral crystallinity (splitting factor) and carbonate to phosphate (${CO}_{3}^{2-}{/PO}_{4}^{3-}$) substitution ratios, providing critical information regarding chemical maturation in Matrix Vesicles (MVs) and biomimetic matrices (Mechiche Alami et al. 2017; Mao et al. 2023; Hayann et al. 2025). A key pending challenge in biomineralization concerns the real-time, non-destructive resolution of transient amorphous mineral precursors (e.g., prenucleation clusters and short-lived ACP) confined within nanoscale lipid environments. Time-resolved ATR-FTIR is currently emerging as a crucial tool to capture how localized ion concentration (Ca^2+^, P_*i*_) and membrane-bound enzymes (such as TNAP) cooperatively modulate lipid order to trigger amorphous-to-crystalline phase transitions at the membrane-water interface.

In hydrated conditions, these systems can be studied using attenuated total reflection (ATR) FTIR. By incorporating a polarizer to execute polarized ATR-FTIR measurements, one can evaluate ATR linear dichroism (ATR-LD). This approach measures the absorbance ratio of parallel- and perpendicularly polarized infrared beams to calculate the order parameters (S_θ_) and orientation angles of protein α-helices relative to the bilayer normal, providing detailed structural information regarding protein insertion and organization within lipid bilayers (Tamm and Tatulian 1997).

#### Raman spectroscopy

Raman spectroscopy provides complementary information to FTIR by detecting variations in molecular vibrations associated with changes in molecular polarizability, rather than dipole moment. Because water produces relatively weak Raman signals, the technique is particularly suitable for investigating hydrated biological systems, including membrane-associated proteins, EVs, and biomimetic membrane models (Ahiri 2026).

Protein conformation dynamics can be assessed through Raman bands associated with the protein backbone, particularly the Amide I (~ 1660–1670 cm⁻^1^) and Amide III (~ 1230–1300 cm⁻^1^) regions. The lipid environment can be characterized through carbon–carbon stretching vibrations (Kalisz et al. 2021). This region distinctly separates into all-trans segments (1060–1080 cm⁻^1^) and gauche conformers (1090–1130 cm⁻^1^), correlating directly with CH₂ FTIR spectra to serve as an internal indicator of intramembrane order (Bispo et al. 2025). Furthermore, the spatial resolution afforded by Raman microspectroscopy allows lateral mapping of protein partitioning between liquid-ordered (L_o_) and liquid-disordered (L_d_) microdomains by monitoring specific lipid intensity ratios (I₁₀₆₂/I₁₀₈₉), simulating the behavior of lipid rafts and receptor clustering observed in vivo (Sharma et al. 2012). To distinguish these subtle macromolecular docking events from background signals in the medium, Surface Enhanced Raman Spectroscopy (SERS) utilizes Localized Surface Plasmon Resonance (LSPR) on nanostructured noble metal substrates, amplifying electromagnetic fields by 10⁶ to 10⁸ times and achieving single-molecule level sensitivity to detect transient, non-covalent structural fluctuations in dilute lipid monolayers (Lim and Kumar 2024).

In biomineralization studies, Raman spectroscopy is valuable for identifying calcium phosphate precursor phases and monitoring mineral maturation. Transient mineral species such as ACP and OCP can be detected through characteristic phosphate bands near 1007–1010 cm^−1^, whereas the formation of crystalline biological apatite is associated with the emergence of the symmetric phosphate stretching mode near 960 cm^−1^ (Mechiche Alami et al. 2017; Mao et al. 2023; Nogueira et al. 2025; Melo et al. 2026).

### Monitoring enzymatic activity and mineralization processes

Beyond structural and compositional analyses, understanding biomineralization requires techniques capable of monitoring the functional activity of osteogenic proteins and the chemical changes occurring at mineralizing interfaces. Processes such as enzymatic Pi generation, ion transport, and mineral nucleation are inherently dynamic and therefore require analytical approaches capable of providing real-time information on protein function and local chemical environments (Simão et al. 2013; Andrilli et al. 2023; Favarin et al. 2025). These methodologies complement imaging and spectroscopic techniques by assessing and monitoring molecular events that drive mineral formation in both native and biomimetic membrane systems.

#### Boron-doped diamond microelectrodes

Boron-doped diamond (BDD) and boron-doped nanocrystalline diamond (BNCD) microelectrodes have emerged as promising electrochemical platforms owing to their wide electrochemical window, high chemical stability, and resistance to corrosion and surface fouling. These properties enable sensitive and reproducible measurements in complex biological environments (Lawrence et al. 2006; Hébert et al. 2014). Unlike conventional ion-selective electrodes, BNCD devices do not require selective membranes, allowing the evaluation of ion fluxes, buffering effects, and protein–membrane interactions (Hébert et al. 2014).

The nanocrystalline structure of BNCD increases the effective electroactive surface area while preserving the intrinsic stability of diamond materials. Combined with the advantages of microelectrode geometries, including enhanced radial diffusion and reduced capacitive effects, these devices provide high spatial and temporal resolution for electrochemical monitoring (Pasquarelli et al. 2022).

Studies using BNCD microelectrodes and annexin-containing proteoliposomes demonstrated the ability of these platforms to monitor Ca^2+^ transport across biomimetic membranes and investigate membrane-associated events involved in early mineralization events (Bolean et al. 2015; Pasquarelli et al. 2022; Ferreira et al. 2023). Together, these features establish BNCD microelectrodes as a valuable tool for investigating transmembrane transport, protein-lipid interactions, and the functional activity of osteogenic proteins in both native vesicles and biomimetic membrane systems.

## Advanced and complementary biophysical approaches and future perspectives

Although considerable advances have been achieved through the methodologies discussed in previous sections, advanced and complementary biophysical approaches have been achieved through the methodologies discussed in previous sections, emerging and complementary techniques continue to expand the analytical toolbox available for investigating membrane-associated biomineralization processes. Established methods such as static light scattering (SLS), small-angle X-ray scattering (SAXS), small-angle neutron scattering (SANS)**,** surface plasmon resonance (SPR) and microfluidic platforms provide valuable information regarding supramolecular organization, membrane architecture, protein-lipid interactions, and processes occurring at mineralizing interfaces. Their application and integration into studies of extracellular vesicles, lipid interfaces, and biomimetic systems continue to expand the analytical capabilities available for investigating membrane structure, protein–lipid interactions, and membrane-associated processes (Varga et al. 2014; Scattarella et al. 2021; Huang et al. 2026). Among these approaches, SAXS enables the investigation of nanoscale membrane organization, lipid packing, and structural rearrangements induced by protein incorporation, whereas SANS provides complementary structural information through neutron scattering contrast. The combination of SAXS and SANS can therefore provide a more comprehensive characterization of nanoscale organization in complex biological and biomimetic systems, particularly when different structural components exhibit distinct neutron and X-ray scattering contrasts (Törnquist et al. 2020). SLS contributes to the characterization of higher-order assemblies and aggregation phenomena. SPR allows real-time, label-free analysis of binding kinetics and membrane association processes, providing quantitative information on protein-lipid and protein-matrix interactions. In parallel, advances in microfluidic technologies have enabled the development of increasingly sophisticated biomimetic platforms that reproduce dynamic physicochemical conditions and permit controlled investigation of vesicle-mediated mineralization processes. Together, these approaches complement conventional imaging, spectroscopic, and biochemical methodologies by providing structural, kinetic, and physicochemical information across multiple length and time scales. Their continued integration with established analytical techniques is expected to contribute to a more comprehensive understanding of membrane-associated biomineralization and to facilitate the development of biomimetic systems and vesicle-based therapeutic strategies (Castellana and Cremer 2006; Homola 2008).

Super-resolution microscopy techniques, particularly stochastic optical reconstruction microscopy (STORM), have expanded the ability to investigate MVs and lipid nanoparticles (LNPs) beyond the diffraction limit of conventional fluorescence microscopy. By sequentially localizing individual fluorophores and computationally reconstructing the final image, STORM achieves spatial resolutions of approximately 20–50 nm, enabling nanoscale visualization of vesicle morphology, protein distribution, and molecular organization at the single-particle level (Rust et al. 2006). The continued development of these approaches is expected to further improve the characterization of MVs and biomimetic membrane systems, providing new opportunities to investigate vesicle biogenesis, molecular composition, membrane dynamics, and interactions with extracellular matrix components. Such advances are particularly relevant because mineralized tissues arise from highly coordinated cellular and extracellular events that regulate matrix organization and mineral deposition (de Wildt et al. 2019; Mansoorifar et al. 2020). Additionally, emerging techniques such as surface plasmon resonance (SPR) and single-particle tracking (SPT) further expand the analytical toolbox for studying osteogenic protein–lipid interactions. SPR enables real-time, label-free analysis of binding kinetics and membrane association processes, while SPT allows direct visualization of diffusion behavior, membrane mobility, and dynamic remodeling events within heterogeneous membrane systems (Castellana and Cremer 2006). Collectively, integrating multiple biophysical and imaging approaches provides a multidimensional strategy for investigating the structural, dynamic, and physicochemical mechanisms governing membrane remodeling and matrix vesicle-mediated biomineralization (de Wildt et al. 2019; Mansoorifar et al. 2020).

A deeper understanding of these mechanisms may accelerate the development of next generation osteoinductive and osteoconductive biomaterials, as well as novel therapeutic strategies based on native MVs and biomimetic vesicular systems. Elucidating how vesicle composition, membrane organization, and protein activity regulate mineral formation may contribute to the design of materials capable of promoting tissue regeneration while also providing new approaches for preventing or treating pathological mineralization. Ultimately, the integration of cell biology, membrane biophysics, materials science, and nanotechnology will be essential for translating fundamental discoveries in biomineralization into clinical and technological applications.

Finally, several important questions regarding MV biology remain unresolved. For example, it is still unclear whether MV age influences their mineralization capacity, whether older and newly released MVs differ primarily in their mineralization potential or signaling functions, and whether TNAP-negative MVs, recently identified by Hayann et al. (2026), exhibit properties comparable to those of TNAP-positive MVs. Addressing these questions is currently limited by the lack of reliable approaches to isolate, purify and characterize MVs according to their age or specific molecular markers. The development of biophysical and analytical approaches capable of resolving such MV heterogeneity may therefore provide important insights into the mechanisms governing MV-mediated mineralization.

## Data Availability

No datasets were generated or analysed during the current study.
